# Monoclonal Antibody Switching in Biologic Treatment of Chronic Rhinosinusitis with Nasal Polyps

**DOI:** 10.3390/jcm13226883

**Published:** 2024-11-15

**Authors:** Michael Habenbacher, Ulrich Moser, Oliver Hadl, Peter Kiss, Clemens Holzmeister, Jakob Pock, Katharina Walla, Angelika Lang, Alexandros Andrianakis

**Affiliations:** Department of Otorhinolaryngology, Medical University of Graz, 8010 Graz, Austria

**Keywords:** biologics, dupilumab, mepolizumab, omalizumab, chronic rhinosinusitis, CRSwNP

## Abstract

**Objectives:** to evaluate our real-world data on the efficacy and safety of switching between two monoclonal antibodies in biologic treatment of uncontrolled chronic rhinosinusitis with nasal polyps (CRSwNP). **Methods:** All patients receiving biologic treatment for uncontrolled CRSwNP between April 2020 and March 2024 at a tertiary referral center who needed transitioning between biologic agents were retrospectively analyzed. The following parameters were investigated: patient’s clinical characteristics, wash-out periods, treatment outcome, and switching-related side effects. **Results:** Out of 91 CRSwNP patients who received biologic treatment, 4 patients (4.4%) necessitated switching to an alternative biologic agent. Three patients were switched to a different antibody because of insufficient symptom control with the initially prescribed biologic, while the other patient required switching to an alternative agent due to a side effect. Switching resulted in all four patients achieving a significant improvement in all outcome parameters. There were no switching-related side effects, and the switching procedure was performed in three cases without a wash-out period. **Conclusion:** CRSwNP patients under biologic therapy with an insufficient response or medication-related side effect may benefit from switching to an alternative biologic agent. Larger, prospective multicenter studies are warranted to further validate the effectiveness and safety of switching agents in the biologic treatment of CRSwNP.

## 1. Introduction

The substantial global impact of chronic rhinosinusitis with nasal polyps (CRSwNP) is widely acknowledged, affecting approximately 2–4% of the Western population [1,2,3]. For CRSwNP, endoscopic sinus surgery (ESS) and oral corticosteroid (OCS) therapy, whether local or systemic, have been traditionally the primary treatment modalities. CRSwNP typically exhibits a type 2 inflammatory profile in the Western population. This inflammation pattern is characterized by heightened levels of IL-4, IL-5, and IL-13, accompanied by eosinophilic infiltration. CRSwNP often presents with comorbidities such as asthma and NSAID-exacerbated respiratory disease (NERD) [4].

Recent therapeutic advancements in chronic rhinosinusitis with nasal polyps (CRSwNP) have introduced biologic medications, including monoclonal antibodies targeting key inflammatory mediators IL-4Rα, IgE, and IL-5, notably dupilumab, omali-zumab, and mepolizumab, respectively. These biologics have expanded treatment options for challenging CRSwNP cases, with established guidelines for prescription and evaluation [1].

However, uncertainties persist regarding personalized antibody selection, the process of switching between biologics in cases of inadequate disease control or adverse effects, and the necessity of wash-out periods. There remains insufficient guidance regarding selection of the appropriate antibody [1]. Real-world data on the efficacy and safety of switching between monoclonal antibodies in the biologic treatment of severe, uncontrolled CRSwNP are scarce [5].

Therefore, we report our real-life experience of switching biologic agents in severe CRSwNP patients, aiming to contribute to a more comprehensive knowledge of biologic treatment of CRSwNP.

## 2. Materials and Methods

### 2.1. Study Design

A retrospective chart review on patients diagnosed with CRSwNP who were treated with biologics at the Department of Otorhinolaryngology of a tertiary referral center between April 2020 and March 2024 was conducted. Diagnosis of CRSwNP was defined by using EPOS criteria [1]. Those patients who had undergone transitioning between biologic agents were included in this study.

### 2.2. Criteria for Biologic Treatment

The prescription of biologic treatment for CRSwNP at our department followed the following EPOS 2020 criteria [1]: (1.) presence of nasal polyps, (2.) previous endoscopic sinus surgery, and (3.) at least three out of five criteria: evidence of type 2 inflammation (blood EOS ≥ 0.25 × 10^9^/L or total IgE ≥ 100 kU/L), need of ≥2 courses systemic COS per year, sinunasal outcome test—22 (SNOT-22) score ≥ 40, impaired sense of smell, and comorbid asthma requiring regular inhaled corticosteroids.

### 2.3. Clinical Assessment

A patient’s characteristics and medical history (age, sex, number of previous ESS, comorbidities including asthma, NERD, and allergy) were recorded during an indication visit. A clinical examination was carried out before beginning biologic treatment (7–14 days prior to start) and every six months thereafter. Clinical evaluation included the following: nasal endoscopy to determine nasal polyp size, SNOT-22, and a blood test evaluating total serum immunoglobulin E (IgE) and eosinophil count (EOS). Nasal polyp size was assessed by using the Meltzer Nasal Polyp Score (NPS) [6].

### 2.4. Evaluation of Treatment Response

Response to a biologic treatment was evaluated six months after the start of therapy. If a switch in the biologic agent occurred earlier or in between follow-up visits, assessment of the treatment outcome was performed six months after initiation of the new biologic agent. The degree of response in our case series was determined by using modified EUFOREA response criteria [7]: (1.) reduced NPS: yes—if NPS reduction ≥ 1; (2.) improved quality of life: yes—if SNOT-22 reduction ≥ 12 points; (3.) improved sense of smell: yes—if SNOT-22 item “Impaired smell” reduction ≥ 1 point; (4.) reduced impact of asthma bronchiale: yes—if asthma control test > 3 points improvement, or >20 points or subjective significant improvement recorded in the patient’s anamnesis; and (5.) reduced need for OCS/salvage ESS: yes—if not needed.

## 3. Results

In total, 91 patients received biologic treatment for CRSwNP at our department between April 2020 and March 2024. The vast majority were treated solely with dupilumab (*n* = 80, 88%). Omalizumab and mepolizumab were exclusively used in six patients (6.5%) and one patient (1.1%), respectively. Four patients (4.4%) necessitated switching to an alternative biologic agent owing to self-reported insufficient symptom control or adverse reactions to therapy. Due to the small number of patients requiring a change in biologic agent, they were analyzed in a descriptive manner. The demographics and medical history of these patients are presented in Table 1 while detailed initial clinical parameters and treatment response results after switching of the biologic agent are presented in Table 2.

### 3.1. Case 1

The first patient (female, 54 years) received omalizumab as a primary therapy. On request, omalizumab as a biologic agent was chosen at that time by the attending consultant due to an elevated IgE count (total IgE 307 kU/L). At the first 6-month follow-up, an initial SNOT-22 score of 80 and NPS of 6 decreased significantly to 4 and 2, respectively. After 18 months of omalizumab treatment, the patient presented to our clinic due to progressive deterioration of symptoms, including nasal congestion, frontal pain, and post-nasal drip. (NPS of 3; SNOT-22 was not assessed). Biologic treatment was switched immediately to dupilumab without a wash-out period, and was free of complications. Six months after the start of dupilumab treatment, the patient presented an excellent response (SNOT-22: 3, NPS: 0) and is currently at a 20-month dupilumab-treatment duration.

### 3.2. Case 2 

The second patient (female, 52 years) received dupilumab as a first-line agent. After 4 months of treatment, dupilumab was discontinued due to joint paint (clinical data on NPS and SNOT-22 not assessed at that time point) and was switched after a 3-month wash-out period to mepolizumab without complications. Mepolizumab was chosen due to an elevated blood EOS (0.4 × 10^9^/L) and normal-ranged total IgE (39 kU/L). At the first 6-month post-mepolizumab follow-up visit, the baseline SNOT-22 score of 58 and NPS of 7 exhibited significant reductions to 23 and 2, respectively. Currently, the patient has completed 16 months of mepolizumab therapy.

### 3.3. Case 3

Our third patient (female, 54 years) also received dupilumab as a first-line biologic. During the first six months, she experienced insufficient sinunasal symptom control and worsening of asthma symptoms. The initial NPS of 2 remained unchanged and SNOT-22 decreased from 46 to 34. Therefore, dupilumab was switched without a wash-out period to omalizumab. Omalizumab was selected due to the presence of polyvalent allergic rhinitis and a resulting elevated total IgE. At the 6-month post-omalizumab visit, she showed a good response (SNOT-22: 34 to 16, NPS: 2 to 0, subjective reduced impact of asthma) and has had 12 months of treatment so far.

### 3.4. Case 4

The fourth patient (female, 56 years) started biologic treatment with mepolizumab. Upon request, the attending consultant selected mepolizumab as the biologic agent at that time because of an elevated blood EOS (0.4 × 10^9^/L). At the first follow-up visit, the patient reported no improvement in her symptoms; indeed, a marginal exacerbation was noted. The NPS and SNOT-22 worsened from initial 6 and 43 to 8 and 60, respectively. Treatment was switched without a wash-out period to dupilumab, and was free of side-effects. Six months after the start of dupilumab treatment, the patient presented an excellent response (NPS: 0, SNOT-22: 30).

## 4. Discussion

Biological therapy has been demonstrated to be effective and safe in the treatment of severe, uncontrolled CRSwNP with or without comorbid asthma [8,9,10]. However, it still remains unclear which biologic agent is best suited for each individual patient. Current evidence guiding the choice of biologic agents for CRSwNP remains limited, yet emerging data provides some direction for individualized treatment selection. A few systematic reviews and a meta-analysis indirectly compared different biologic agents and reported dupilumab to be the most effective antibody for CRSwNP [11,12,13]. Moreover, available studies indicate that patient characteristics, such as comorbid asthma, eosinophil count, and total IgE levels, play a critical role in determining the optimal biologic therapy. For instance, patients with both CRSwNP and asthma may benefit more from dupilumab as several studies have demonstrated its superior efficacy in managing both nasal and lower respiratory symptoms when compared to anti-IgE or anti-IL-5 therapies, which often show inadequate upper airway control [14,15]. On the other hand, omalizumab may be more appropriate in patients with elevated total IgE or those with co-existing allergic disease [16,17], while mepolizumab may be better suited for patients with high eosinophil counts [18]. 

Certainly, the choice might also be influenced by the emergence of biologic treatments in the market, along with the preferences of healthcare providers and insurers. Dupilumab was the first available biologic medication licensed for CRSwNP in 2019, followed by omalizumab in 2020, and mepolizumab in 2021. The vast majority of our CRSwNP patients were treated with dupilumab due to several reasons: here in Austria, a prescription for dupilumab by health insurance companies is easier compared to the other antibodies; earlier approvement and associated familiarity with the medication; rising real-world superiority in efficacy [5,11,12,13]. However, in real-world practice, the costs of biologic therapies also pose a significant consideration. In Austria, the annual costs per patient for dupilumab, mepolizumab, and omalizumab are approximately 13.824 €, 11.619 €, and 8.166 € (sales price excluding sales tax), respectively [19]. This substantial cost difference requires a critical evaluation of economic factors when selecting biologic therapies. A recent Canadian study has identified omalizumab as the most cost-effective option among biologics for treating CRSwNP [20]. Although dupilumab has demonstrated superior efficacy in several studies [12,13], omalizumab’s broader indications, including its efficacy in chronic idiopathic urticaria [21] and its recent approval for multiple food allergies, provide further valid reasons for its use [22,23]. Moreover, omalizumab’s much longer market presence since 2003 (compared to mepolizumab in 2015 and dupilumab in 2017) ensures a robust and well-documented safety profile, which is vital for long-term treatment considerations [23]. 

The initial choice of biologic leads in most CRSwNP cases to a very favorable therapeutic response when adhering to the indication criteria. In a minority of patients, the first biological drug treatment does not result in controlled symptoms, or the medication causes significant side effects, necessitating a switch to an alternative antibody. Real-world data on switching biologics in the treatment of severe, uncontrolled CRSwNP are limited. In a recent multi-center study, Otten et al. [14] analyzed the transitioning of different biologics in 94 patients with comorbid CRSwNP and asthma who experienced inadequate upper and/or lower air tract symptom control. The authors described the different combinations of changes (e.g., mepolizuumab->dupilumab; rezlizumab->omazilumab), and that, in some cases, more than just one switch was necessary (e.g., omalizumab->mepolizumab->dupilumab). While the authors aimed to propose an algorithm to assist in selecting optimal therapeutic management for non-responsive cases, they predominantly offered generalized explanations of favorable or unfavorable biologic responses and did not provide an explanation for their choice of biologics. Another recent study evaluated 20 patients with coexisting CRSwNP and asthma who were switched from omalizumab, mepolizumab, or benralizumab to dupilumab due to insufficient nasal symptom control. The authors reported an overall good response to dupilumab; however, they did not specify why dupilumab was always chosen as an alternative agent, and they did not report the rate of non-responders to dupilumab as a first-line choice [24]. In the present case series, all four cases of different transitioning patterns in biologic treatments resulted in efficient symptom control. We tried to select the most appropriate alternative biologic agent based on potential predictors of response. In case 2, mepolizumab was chosen due to an elevated blood EOS, normal-ranged total IgE, and no history of allergic disease. In case 3, omalizumab was selected due to the presence of polyvalent allergic rhinitis and a resulting elevated total IgE. In the other two cases, no rationale for the choice of the alternative agents was documented unfortunately. However, as previously mentioned, dupilumab was frequently selected at our department due to its earlier approval alongside higher familiarity, rising real-world superiority in efficacy [11,12,13], and ease of prescription through health insurances in Austria. These factors most likely influenced the choice of dupilumab as an alternative biologic in these two cases.

Recent studies provide further insights into the practice of switching between biologics in asthmatic patients with or without CRSwNP. Higo et al. [25] conducted a multi-center retrospective study examining the effects of switching to dupilumab from other biologics, including omalizumab and mepolizumab, without a treatment interval in patients with severe type 2 asthma. This study demonstrated that switching to dupilumab can be performed safely and effectively, resulting in improved asthma control and patient outcome. Similarly, Carpagnano et al. [26] presented a real-life experience from Southern Italy, where patients with severe eosinophilic allergic asthma, uncontrolled by omalizumab, were switched to mepolizumab. This study found significant improvements in asthma control, lung function, and a reduction in exacerbations, highlighting the therapeutic benefit of switching to mepolizumab in patients who do not respond adequately to omalizumab.

Out of all CRSwNP patients receiving biologic therapy at our clinic, only one patient required switching to an alternative biologic agent due to a side effect, namely joint pain. Similar results were also reported by Brkic et al. [5] in which one patient out of 195 changed biologics due to an adverse reaction to dupilumab. According to a recent real-world study, arthralgia is the most common side effect of dupilumab at 16% [27]. In severe cases of side effects, switching to mepolizumab or omalizumab might be necessary. 

Requirement for a wash-out phase preceding the initiation of a subsequent biologic therapy remains a topic of uncertainty [28]. The current literature advocates for a wash-out duration of 5–10 half-lives or approximately 3 months [29]. Given that the half-lives of biologics used in the management of CRSwNP fall within the range of 15 to 26 days, enforcing a wash-out period may carry the risk of exacerbating symptoms significantly, thereby adversely affecting patients’ quality of life. Consequently, an immediate switch between biologics could offer potential benefits for patient well-being. Papaioannou et al. [30] reviewed available data on switching biologics in patients with severe type 2 asthma and proposed that transitioning between monoclonal antibodies in asthma treatment is safely feasible without a wash-out period. Recently, Brkic et al. [5] reported that approximately 50% of their CRSwNP patients (*n* = 23) who required transitioning between biologic agents were successfully switched to an alternative medication without a wash-out period. Initially, the authors aimed to switch the biologic drug in all their patients without a wash-out phase, but this was often not possible due to the COVID-19 pandemic. We observed similar results in our case series. The biologic agent was changed in three out of four cases (75%) immediately without a wash-out period, and no adverse effects occurred. In the case of the patient with biologic-related joint pain, a wash-out period of 3 months was initiated before the new biologic agent was started to avoid the possibility of an additional side effect.

All four patients in our study were females. According to previous trials, there are no significant sex differences in the outcome of biologic treatment in CRSwNP patients [8,9,10,31]. However, it is important to note that some studies have reported conflicting conclusions regarding sex differences in CRSwNP characteristics including disease burden and comorbidities [32,33,34,35,36,37]. In particular, females may exhibit a higher burden of disease [32,33,34,35] and higher prevalence of comorbid asthma and NERD [32,36]. Despite these potential differences, the overall efficacy of biologic treatment remains consistent across sexes [8,9,10,32].

In our study, all patients requiring biologic transitioning were in their fifth decade of life, which is consistent with the general age distribution observed in CRSwNP patients requiring biologic treatments [8,9,10,11,12,13]. Previous population-based studies have also shown that the peak prevalence of CRSwNP occurs in individuals aged 50–60 years [2,38,39]. The need for more aggressive treatment strategies, such as biologics, might increase with age due to the cumulative burden of chronic inflammation and comorbid conditions. Our findings, therefore, align with established age-related trends in CRSwNP management.

There are several limitations to this study that must be acknowledged. First, the small sample size, coupled with the single-center setting, limits the generalizability of our findings to a broader population. Second, the retrospective and observational nature of the study inherently increases the risk of selection bias and the presence of unmeasured confounding factors. Additionally, the lack of standardized olfactory testing at our institution meant that we had to rely on patient-reported data regarding sense of smell for both indication and response assessment. Nevertheless, with the present case series, we contribute to a more comprehensive knowledge of switching agents in the biologic treatment of CRSwNP.

## 5. Conclusions

Three of our four patients were switched to an alternative biologic agent because of insufficient symptom control with the initially prescribed biologic, while the other patient required switching to a different antibody due to an adverse reaction. The switching procedure was well-tolerated and deemed safe, with no side effects noted. Furthermore, switching resulted in effective symptom control for patients, even without implementing a wash-out period. CRSwNP patients under biologic treatment with an inadequate response or medication-related side effect may benefit from switching to an alternative biologic agent. Larger, prospective multicenter cohort studies are warranted to further validate the effectiveness and safety of switching agents in the biologic treatment of CRSwNP.

## Figures and Tables

**Table 1:** Clinical characteristics of patients switching biologics.

	Case 1	Case 2	Case 3	Case 4
Biologic Switch	Oma->Dupi	Dupi->Mepo	Dupi->Oma	Mepo->Dupi
Indication for switching	Insufficient symptom control	Joint pain	Insufficient symptom control	Insufficient symptom control
Age (years)	54	52	54	56
Sex	f	f	f	f
No. of previous ESS	1	2	2	3
Allergic rhinitis	yes	no	yes	yes
NERD	no	no	yes	yes
Comorbid asthma	no	yes	yes	yes

ESS: endoscopic sinus surgery; NERD: NSAID-exacerbated respiratory disease.

**Table 2:** Indication criteria for biologic treatment and response results after switching biologics.

	Case 1 Oma->Dupi	Case 2 Dupi->Mepo	Case 3 Dupi->Oma	Case 4 Mepo->Dupi
Initial NPS	6	7	2	6
No. of EPOS indication criteria	4/5	4/5	3/5	4/5
Blood EOS (10^9^/L)	0.4	0.4	0.5	0.4
Total IgE (kU/L)	307	39	127	280
Need for OCS	yes	no	no	no
SNOT-22	84	57	46	43
Smell function	5 points on SNOT-22 smell-subdomain	4 points on SNOT-22 smell-subdomain	0 points on SNOT-22 smell-subdomain	4 points on SNOT-22 smell-subdomain
Comorbid asthma	no	yes	yes	yes
EUFOREA response criteria 6 months after switching	4/5	5/5	4/5	5/5
NPS	2	2	0	0
Improved QOL (SNOT-22)	3	23	16	30
Improved sense of smell	0 points on SNOT-22 smell-subdomain	3 points on SNOT-22 smell-subdomain	0 points on SNOT-22 smell-subdomain	3 points on SNOT-22 smell-subdomain
Reduced impact of comorbidities	n.a.	yes	yes	yes
Need for OCS/salvage ESS	no	no	no	no

Modified EUFOREA response criteria: reduced NPS: yes—if NPS reduction ≥ 1, no—if NPS reduction < 1; improved QoL: yes—if SNOT-22 reduction ≥ 12 points, no—if SNOT-22 reduction < 12 points; improved sense of smell: yes—if SNOT-22 item “Impaired smell” reduction ≥ 1 point, no—if SNOT-22 item “Impaired smell” reduction < 1 point; reduced impact of comorbidities: yes—if asthma control test (ACT) improved by ≥3 points, or ≥20 points or subjective significant improvement, no—if ACT improved by <3 points, or <20 points or if ACT not available—no subjective improvement; NPS: nasal polyp score; OCS: oral corticosteroids; EOS: eosinophils; ACT: asthma control test; SNOT-22: sinunasal outcome test 22; QOL: quality of life, EPOS: European Position Paper on Rhinosinusitis and Nasal Polyps; ESS: endoscopic sinus surgery; n.a.: not applicable/available.

## Data Availability

The original contributions presented in the study are included in the article; further inquiries can be directed to the corresponding author.
